# How behavioral economics can help to avoid ‘The last mile problem’ in whole genome sequencing

**DOI:** 10.1186/s13073-015-0132-8

**Published:** 2015-01-22

**Authors:** Jennifer S Blumenthal-Barby, Amy L McGuire, Robert C Green, Peter A Ubel

**Affiliations:** Center for Medical Ethics and Health Policy, Baylor College of Medicine, One Baylor Plaza MS 420, Houston, TX 77030 USA; Division of Genetics, Department of Medicine, Brigham and Women’s Hospital and Harvard Medical School, Avenue Louis Pasteur, Boston, MA 02115 USA; Fuqua School of Business and Sanford School of Public Policy, Duke University, Fuqua Drive, Durham, NC 27708 USA

## Abstract

Failure to consider lessons from behavioral economics in the case of whole genome sequencing may cause us to run into the ‘last mile problem’ - the failure to integrate newly developed technology, on which billions of dollars have been invested, into society in a way that improves human behavior and decision-making.

Much energy and other resources are being invested in efforts to integrate whole genome sequencing (WGS) and whole exome sequencing (WES) into clinical care. For example, the National institutes of Health (NIH) plans to invest $400 million into translational genomic projects over the next few years. Already, millions of dollars have been committed to exploring the clinical integration of genomics through programs such as the Clinical Sequencing Exploratory Research (CSER) Consortium. This consortium funds nine centers to study the generation of genomic sequence data, methods to translate and interpret those data, and the ethics and psychosocial impact of those data in a variety of pediatric and adult clinical settings. As members of one of the funded centers, we are optimistic about the future of clinical genomics. Nevertheless, in addition to well-recognized technical and translational challenges that are associated with the clinical integration of WGS or WES, the field of behavioral economics suggests that there will be equally daunting behavioral challenges.

Behavioral economics is a field of study which emphasizes that provision of information does not necessarily lead to rational use of information. Failure to consider lessons from behavioral economics in the case of WGS or WES may cause us to run into what economist Sendhil Mullainathan calls ‘the last mile problem’ [1]. This is the investment of billions of dollars in resources to develop new technology, but the failure to integrate that technology into society in a way that improves human behavior and decision making. The aim of this comment is to outline how several cognitive biases that are well-studied in behavioral economics - information-seeking bias, affect bias, and impact bias - might impede the successful integration of WGS and WES into clinical care and to suggest strategies for mitigating them. In doing so, we highlight the importance of considering these issues as part of ongoing research. To our knowledge, our group is the only project explicitly examining behavioral economics issues and their impact on translational genomic research. We believe that these considerations will be integral to the success of clinical genomics.

## Information-seeking bias and defaults

One potential example of the last mile problem can be found in a phenomenon that behavioral economists call ‘information-seeking bias’ - the tendency people have to seek information even if that information lacks utility or meaning. In the context of genetic testing, surveys show that many people report that they want all genetic results returned, even if the meaning of those results is unknown [2]. It has been suggested that disclosing ‘all of the information’ , even when much of it is ambiguous, could lead to wasted healthcare resources and iatrogenic harms if additional diagnostic tests or procedures were pursued. In our own study (the MedSeq Project) [3], we have found patients who expressed intentions to deal with any ambiguous genetic information by seeking second opinions and referrals to other clinicians. The collection of post-disclosure data to examine patients’ actual behaviors regarding second opinions and referrals is ongoing. At 6-months follow-up, 6 of 18 participants have reported sharing their genetic information (disclosed by their doctor) with another doctor (ALM, JBB, PU and RG, unpublished data).

To help mitigate information-seeking bias and its effects in genomic medicine, clinicians could decide to provide patients with a specific information set (for example, results they deem to be actionable and of clinical utility) as the ‘default’ , while allowing patients to opt out if they want more extensive results returned. For our clinical sequencing exploratory research study (the MedSeq Project), participating genetics experts judged that the default set of results returned should include information about: (1) high-penetrance disease mutations (such as those related to hypertrophic cardiomyopathy), (2) disease-risk alleles (for example, variants for prostate cancer or venous thromboembolism), (3) their carrier status for highly penetrant recessive diseases (such as cystic fibrosis and Tay-Sachs disease), and (4) pharmacogenomic traits (such as an increased sensitivity to warfarin). Other experts may come to different judgments. But even this preliminary judgment raises two important issues. First, some patients may perceive this approach to be overly paternalistic. Yet, under this approach, patients remain free to receive the information they desire while being more likely to focus on the most relevant information. Numerous studies in behavioral economics have demonstrated the power of defaults in improving people’s decisions [4]. Second, the use of defaults raises the challenge of deciding what to include in the default group of results. In part, this challenge can be met through a fair and multi-stakeholder process, as is often the case with the establishment of best-practice standards surrounding contentious issues.

## Affect bias and contextualization of risk

Another important behavioral economics concept relevant to WGS and WES integration is the ‘affect bias’ , the finding that risk perceptions and related behaviors are influenced more by people’s emotional response to risk than by their cognitive evaluation of that risk [5]. Emotional responses to risk such as fear and anxiety are often influenced less by the magnitude of the risk (for example, 1 in 10,000 versus 1 in 100,000) than by the nature of the risk itself (for example, an elevated cancer risk might provoke an especially strong response) [5]. WGS and WES will deliver a lot of information to patients about their health risks, and patients may demonstrate oversensitivity to information on the possibility of developing diseases that is not tempered by the associated probability of disease occurrence [5]. This could be compounded by the inaccurate perception that all genetic risks are deterministic.

In many settings, it will be crucial to counter these reactions by contextualizing and framing genetic risk information for patients. Behavioral economists have found that response to risk information can be improved by using various contextualization techniques [6]. Contextualization of risk includes giving patients comparative risk information in addition to information on absolute risk. For example, patients could also be told of competing risks that they would face over the next five years and/or the genetic risk of most other people [6]. Additional methods include presenting risk information in both positive and negative frames and using pictographs to make risk statistics easier to interpret [6].

## Impact bias and behavior-change techniques

An additional behavioral economics concept that is relevant to the clinical integration of WGS and WES is the tendency that people have to inaccurately predict how health conditions or test results will affect their lives. This results from an ‘impact bias’ whereby people overestimate the intensity and duration of emotions brought on by new circumstances [7]. In the context of WGS or WES, patients may seek genetic testing out of a belief that the information will help motivate them to pursue healthier behaviors, predicting that learning about an elevated risk of heart disease, for example, will spur them on to exercise or to go to the doctor for check-ups more regularly. But patients might overestimate the intensity and duration of positive motivational states that are associated with their results. Caulfield and colleagues [8] have linked this to the ‘rhetoric of patient empowerment’ in WGS and WES, noting that there is little evidence to support the premise that patients will use genetic-risk information to improve their lifestyle and reduce their disease susceptibility.

The effects of the impact bias can be countered by taking care to combine the reporting of WGS or WES information with techniques known to promote longer-lasting behavioral change. Behavioral economics has identified several effective ways to help people follow through on their behavioral intentions. For instance, clinicians can utilize the ‘present bias’ by offering small and immediate payments for beneficial behaviors such as smoking cessation, medication adherence or weight loss [9]. In addition, clinicians may be able to overcome the short-lived nature of this motivation by coupling discussion of WGS or WES results with techniques that utilize the desire to follow through with promises and commitments to others. These and other techniques (along with associated ethical considerations) have been outlined elsewhere [10].

The suggestion that behavior changes that are based on WGS or WES risk information should be prescribed or even incentivized may engender concerns about the appropriateness of such practice, given that thousands of common variants provide only modest risk information (typically with relative risk of less than 2.0) [9]. But the ends of behavior change prescriptions to stop smoking, to exercise and to eat healthier are valuable for all patients, regardless of risk, and they are also, in many cases, goals articulated by the patient.

## Conclusions

Understanding human behavior is key to successfully integrating WGS and WES into clinical medicine. Behavioral economics provides us with insights into the less than optimal ways that patients may respond to their WGS or WES information, but it also provides us with insights into how to minimize these suboptimal reactions and how to maximize the positive ways patients can use this information to improve their lives. Funded research should focus on studying these issues and solutions explicitly. Currently funded projects should analyze their data (including audio tapes of disclosure visits, follow-up interviews or surveys with patients who received their WGS or WES results) with an eye to these issues. Future funded research projects should study the effectiveness of strategies to mitigate some of these effects. Without this focus, we risk abandoning the effort to implement genomic medicine before completing the last mile.

## Abbreviations

WES: whole exome sequencing
WGS: whole genome sequencing
